# Counting animals in aerial images with a density map estimation model

**DOI:** 10.1002/ece3.9903

**Published:** 2023-04-07

**Authors:** Yifei Qian, Grant R. W. Humphries, Philip N. Trathan, Andrew Lowther, Carl R. Donovan

**Affiliations:** ^1^ School of Mathematics and Statistics University of St Andrews St Andrews Fife KY169AJ UK; ^2^ HiDef Aerial Surveying Ltd, The Observatory Dobies Business Park Lillyhall Cumbria CA14 4HX UK; ^3^ British Antarctic Survey High Cross, Madingley Road Cambridge CB3 0ET UK; ^4^ Ocean and Earth Science, National Oceanography Centre Southampton University of Southampton University Road Southampton SO17 1BJ UK; ^5^ Norwegian Polar Institute Framsenteret, Postboks 6606, Stakkevollan 9296 Tromsø Norway

**Keywords:** abundance estimation, density map estimation, image processing, machine learning

## Abstract

Animal abundance estimation is increasingly based on drone or aerial survey photography. Manual postprocessing has been used extensively; however, volumes of such data are increasing, necessitating some level of automation, either for complete counting, or as a labour‐saving tool. Any automated processing can be challenging when using such tools on species that nest in close formation such as *Pygoscelis* penguins. We present here a customized CNN‐based density map estimation method for counting of penguins from low‐resolution aerial photography. Our model, an indirect regression algorithm, performed significantly better in terms of counting accuracy than standard detection algorithm (Faster‐RCNN) when counting small objects from low‐resolution images and gave an error rate of only 0.8 percent. Density map estimation methods as demonstrated here can vastly improve our ability to count animals in tight aggregations and demonstrably improve monitoring efforts from aerial imagery.

## Graphical abstract

We present here an adaptation of state‐of‐the‐art crowd‐counting methodologies for counting of penguins (average size is about 5 * 5 pixels) from aerial photography. Our method performed significantly better in terms of model performance and computational efficiency than standard Faster‐RCNN deep learning approaches and gave an error rate of only 0.8 percent.

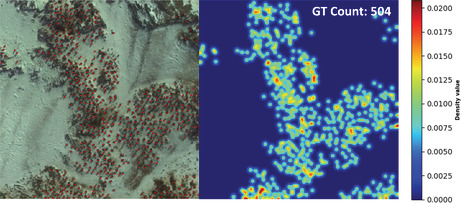

## 1 INTRODUCTION

**FIGURE 1:**
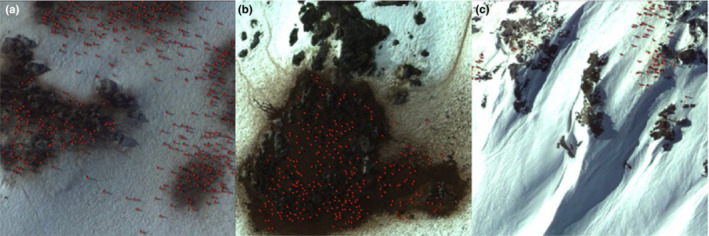
Selected data samples with (a) small object size, (b) almost indistinguishable fore/background objects, and (c) varying illuminations are shown. The study object, penguins, is marked with red dots.

Aerial imagery has become the principal surveying method for many animal populations (Butler & Muller‐Schwarze, 1977; Fraser et al., 1999; Trathan, 2004; Trathan et al., 2012). Such methods are favored since they can quickly and efficiently survey large remote areas with the help of either manned fixed‐wing vehicles/helicopters or unmanned aerial vehicles (UAVs), although the conditions associated with each platform type may dictate subsequent image processing. In the past decades, many such ecological surveys have been conducted (Burn et al., 2006; Chabot et al., 2018; Descamps et al., 2011; Dunstan et al., 2020; Groom et al., 2013; Lee et al., 2019; Vermeulen et al., 2013). While this is a very efficient way to collect large amounts of data, it may create a large postprocessing burden that is frequently borne by humans—typically consisting of laborious manual scanning of photos or videos to locate, identify, and count individual animals (Torney et al., 2016). Volume aside, this can be a challenging task due variously to small object sizes, almost indistinguishable fore/background pixels, and varying illuminations (see Figure 1).

To alleviate these problems, there has been extensive work to integrate computer‐based image processing to assist in, or fully automate, abundance estimation. Early works were mostly based on either spectral thresholding approaches or template matching approaches to count targets of interest in imagery (Chabot & Bird, 2012; Chabot & Francis, 2016; Christiansen et al., 2014). However, those methods are often vulnerable to complex situations such as heterogeneous backgrounds (Chabot & Francis, 2016). Hurford (2017) introduced image processing software, ImageJ to assist in counting birds, but its practical use was limited since the software cannot handle complex environments, which is common in various ecological contexts. To alleviate this problem, Marchowski (2021) preprocessed images with a denoising neural network (Buchholz et al., 2020) before counting with ImageJ, which then makes the counting accuracy highly dependent upon the performance of the denoising process. Object‐based image analysis (OBIA) has also been popular in previous ecological field studies (Afán et al., 2018; Chrétien et al., 2016; Lhoest et al., 2015; Rush et al., 2018). This method first relies on some handcrafted features to group pixels into objects and then classifies objects according to properties such as shape and size, but its counting performance may suffer when animals are obscured or spatial resolution is low (Afán et al., 2018; Chrétien et al., 2016). Hodgson et al. (2016) and Hodgson et al. (2018) offer some other examples of computer‐assisted animal counting, where a combination of Fourier analysis and support vector machines are used to exclude background pixels, making the subsequent manual counting of animals easier. For fully automated estimation of animal numbers, convolutional neural networks (CNNs) are commonly adopted, which are a type of deep learning neural network with components particularly directed toward images. Their use in image processing has been transformative, with robustness proved in classification, detection, and segmentation (Simonyan & Zisserman, 2015).

Automated counting of animals within images usually involves the location, and subsequent classification, of objects within a frame. In terms of CNNs, this gives rise to two broad approaches: one‐ and two‐stage algorithms. Two‐stage algorithms first propose bounding boxes for locations where objects are likely to exist and then do the classification, where region‐based convolutional neural network (RCNNs, Girshick et al., 2014) and Faster‐RCNN (Ren et al., 2016) are representative examples. One‐stage methods such as You Only Look Once (YOLO; Redmon et al., 2016) and Single Shot Multibox Detector (SSD; Liu et al., 2016) process these two tasks simultaneously. In general, one‐stage methods have the advantage of computing speed while two‐stage methods have better accuracy.

Both methods have been adopted for analyzing aerial images collected in ecological studies. Torney et al. (2019) built a YOLO v3 (Redmon & Farhadi, 2018) model to detect wildebeest in aerial images, which displayed accuracy similar to manual processing while being quick to compute. Later, more studies applied YOLO‐based methods to detect their own target species in drone footage (Corcoran et al., 2019; Desai et al., 2022; Gorkin et al., 2020; Hamilton et al., 2020). Another one‐shot object detector, RetinaNet (Lin et al., 2020) was used in an attempt to build a general model for bird detection (Weinstein et al., 2022). Kellenberger et al. (2017) used a Faster‐RCNN model to detect different animals in UAV images surveyed in Kuzikus Wildlife Reserve park. Additionally, the two‐stage Faster‐RCNN model has also been used to detect koalas (Hamilton et al., 2020), kiang (Peng et al., 2020), and large herbivores (Ma et al., 2022) in aerial images. Hong et al. (2019) compared the performance of different deep learning‐based detection methods (Faster‐RCNN, SSD, YOLO, RetinaNet) on a UAV aerial image dataset of wild birds and showed the potential of these techniques in monitoring wild animals. Their study pointed out that the two‐stage method Faster‐RCNN performs the best among all these detection methods with regard to counting accuracy.

Recently, Hoekendijk et al. (2021) proposed a deep CNN model to regress the count objects of interest in the image. Their model is composed of a ResNet (He et al., 2016) and two fully connected layers. Although showing good performance, their model has a size limit on the input images, which means for a large image, it has to be cropped to a required patch size before passing into the model. This may result in issues such as replicated counts across the boundary of these image patches. Also, their results show the model only performs well up to a certain count level—when the count is out of this scope, the model exhibits poor performance.

Here, we adopt a fundamentally different method for counting animals, where the detection of individual animals is avoided, with focus being the estimation of a density map—a concept initially introduced by Lempitsky and Zisserman (2010). Estimated counts are instead obtained by the subsequent integration of this density map, rather than explicit counting of objects. The density map approach has been further integrated into the deep learning framework and widely applied in crowd counting (Lin et al., 2021; Ma et al., 2019, 2021; Qian et al., 2022), where crowds are usually humans.

In this work, we provide a solution to counting animals of low resolution in aerial images by creating a density map estimation model based on CNNs. To demonstrate the superiority of our method, we compare it with the typically used detection method, Faster R‐CNN, which has been found previously to give the most accurate counts among various detection methods (Hong et al., 2019). Our model outperforms the Faster R‐CNN method by a large margin, which has difficulty in detecting very small objects. This is particularly relevant for our exemplar penguin data, where the objects of interest are small in terms of pixels and the performance of detection methods is expected to degrade. Our model also shows robustness when handling images with different object density levels.

## 2 MATERIALS AND METHODS

### 2.1 Data

#### 2.1.1 Data collection

The British Antarctic Survey currently holds an archive of color digital aerial photography from the Antarctic Peninsula and South Shetland Islands acquired between November and December 2013, and partially re‐flown in November 2015. The archive contains images from approximately 140 *Pygoscelis* penguin colonies selected for a range of species, population sizes, and topographic settings. The images were acquired using a large‐format Intergraph DMC mapping camera, with a resolution of about 12 cm or better. The images each have a footprint of about 1600 m × 1000 m and were flown with 60% overlap to allow stereo‐cover. For the images to be useful as part of an automated penguin counting process, they needed significant preprocessing to geolocate them and remove terrain distortions inherent to the perspective view of a camera image. This processing comprised: (1) the stereo‐images were used to extract a digital elevation model (DEM); (2) the images were ortho‐rectified to the DEM to remove terrain effects; (3) the processed images were mosaicked; and then, (4) cut into standard‐sized (448 × 448 pixels) tiles for counting. This process ensures that the images are accurately located and scaled to enable accurate ground area measurements and hence penguin density estimates. Without the DEM and ortho‐rectification preprocessing, the counts would not have a reliable ground area estimate. Stages (3 and 4) also ensure that each penguin only appears once in the dataset. The process to create the DEM is relatively complex and utilized BAE Systems Socet GXP photogrammetry software to generate DEMs, ortho‐rectify the images, and prepare geo‐referenced mosaics for each colony. Aerial imagery from the Intergraph DMC mapping camera allowed multiple penguin colonies to be photographed within a single survey flight on board a deHavilland Twin Otter. This is advantageous when synoptically surveying large areas of terrain where many penguin colonies may occur. *Pygoscelis* penguins generally breed within colonies that comprise a single species, although on occasions there may be two species in close proximity where their colony boundaries interdigitate (Dunn et al., 2021). Our study did not use colonies where two species co‐occur as we only considered separate colonies of gentoo (*Pygoscelis papua*), Ad'elie (*P. adeliae*), and chinstrap (*P. antarctica*) penguins. In contrast, surveys using UAVs may facilitate higher resolution imagery, but operational constraints mean synoptic surveying can be logistically challenging. For the foreseeable future, both types of image capture (light aircraft and UAV) are likely to remain important. Here, we focus upon imagery acquired using the large‐format intergraph DMC mapping camera; future studies will also test the applicability of our methods to higher resolution imagery acquired with UAVs.

#### 2.1.2 Density map generation

Our objective was to estimate the number of penguins in an image, here approached by density map estimation. The density maps are an intermediate representation generated from point annotations, with the integration of any region on these maps providing the count of target objects. The generation process is detailed here.

Given an image I with pixels M and a set of 2D annotated points $P=\left\{p_{1},p_{2},\ldots ,p_{n}\right\}$, its ground‐truth density map $D_{\mathrm{gt}}$ can be obtained by

(1) $$D_{\mathrm{gt}}\left(I_{m}\right)=\sum_{n=1}^{N}N\left(I_{m};p_{n},\sigma_{n}^{2}\right)$$

where $I_{m}$ denotes a two‐dimensional pixel location, m=1,2,…M and $N\left(I_{m};p_{n},\sigma_{n}^{2}\right)$ represents the *n* ^*th*^ annotated two‐dimensional Gaussian distribution, $p_{n}$ is the coordinate of *n* ^*th*^ point annotation, and $\sigma_{n}^{2}$ indicates the isotropic variance. The setting of $\sigma_{n}^{2}$ is flexible and often dataset dependent. It can be either fixed (Lempitsky & Zisserman, 2010) or adaptive (distance to nearest neighbours; Zhang et al., 2016). When using the kernel with fixed bandwidth, we are assuming objects are independently distributed on the image plane, while the adaptive bandwidth is normally used to characterize the geometry distortion led by the perspective effect.

**FIGURE 2:**
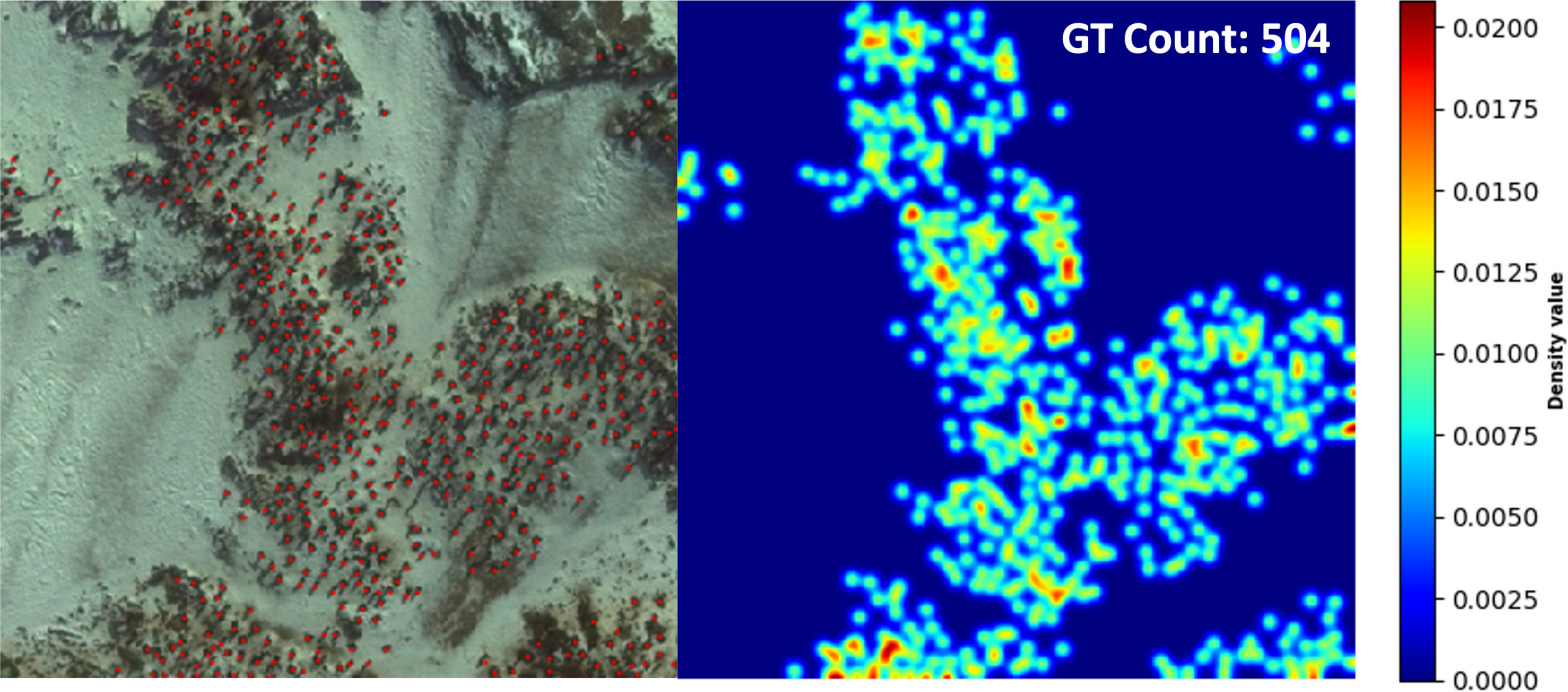
Left is a random image (penguins are labeled with red dots) picked from the dataset and its corresponding density map is on the right.

The choice of $\sigma_{n}^{2}$ is crucial for generating density maps, and using an improperly generated density map as a learning target may compromise the model's counting performance (Wan & Chan, 2019). Ideally, the pixels with density values should reflect consistent features, which in our case means only pixels belonging to a penguin will have density values. However, this is hard to achieve, given the typical size of a penguin is only about 5 × 5 pixels, while using a very small Gaussian kernel will lead to a very unbalanced sparse matrix with most values of 0, and will make the network hard to train (Wang et al., 2020). To achieve the balance, our generation method is given as follows: given the penguins are almost identical in size and shape in aerial images, the Gaussian kernel with fixed bandwidth is applied to the center point of each penguin and the value of *σ* is set as 4. An example of these density maps is given in Figure 2. Although we don't give the location of each penguin, these density maps still retain some location information, which can indicate the region where the penguin may exist.

### 2.2 Specification of the density map estimation model

#### 2.2.1 Model structure

**FIGURE 3:**
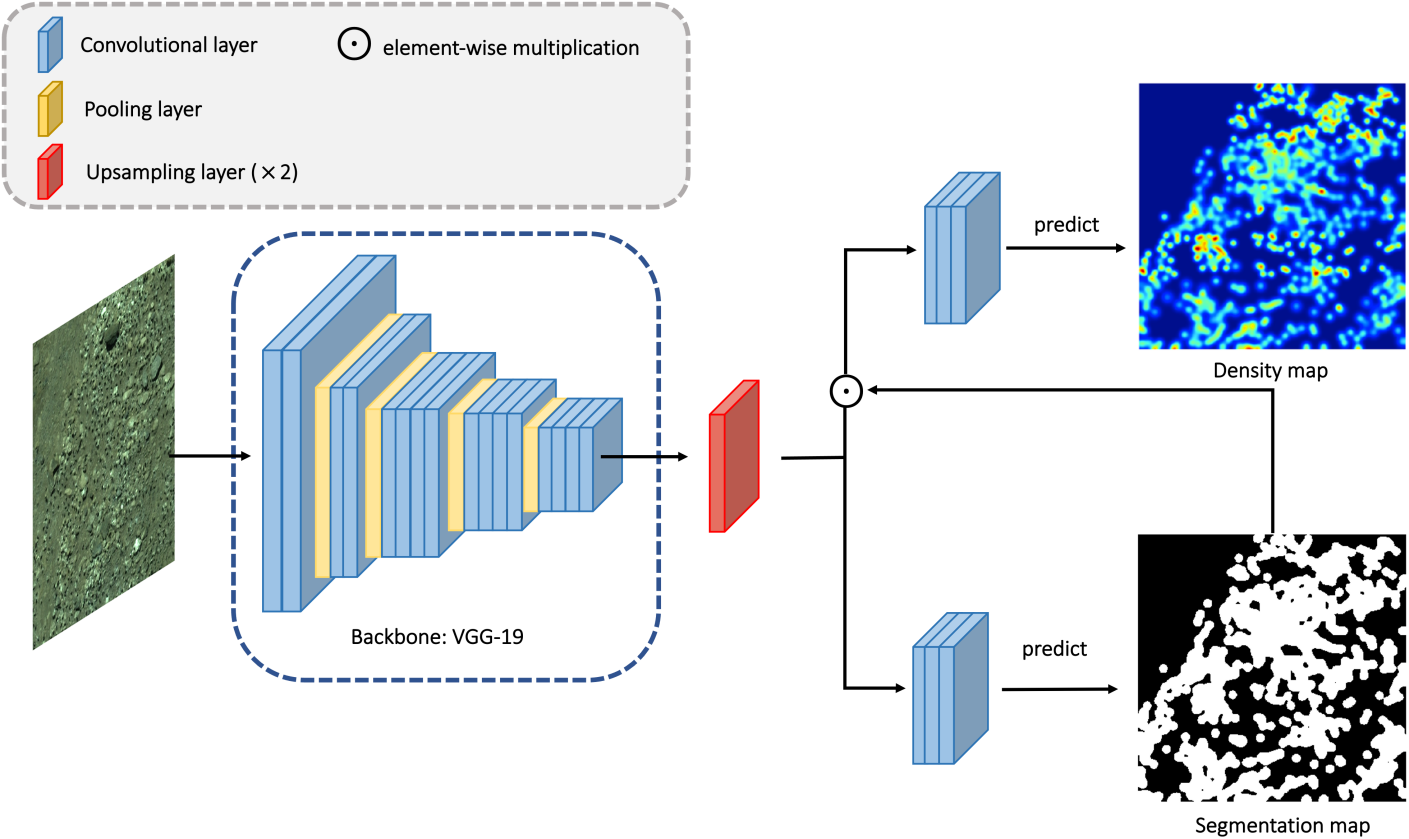
This figure shows the overall structure of our density map estimation model. The backbone extracts features from the input image, and these intermediate features are further fed to two branches to predict density map and segmentation map.

The overall model structure is shown in Figure 3. It is a simple structure with only a backbone network and two branches. Since VGG‐19 (Simonyan & Zisserman, 2015) has good performance in most computer vision tasks, such as detection and classification, and consumes relatively few computing resources, we adopt it as the backbone. However, VGG‐19 learns salient features by gradually downsampling the feature maps. To maintain high resolution of the output density map, we remove its last max pooling layer and all subsequent layers. Additionally, an upsampling layer is added to keep the final size of the output at 1/8 of the original input. Here, bilinear interpolation is used as the upsampling method.

The models are designed to process two tasks: density map estimation and segmentation. Density map estimation can be seen as a two‐step problem by nature, first the location of regions that contains objects of interest and then regress the density values. Second, segmentation is to classify if a pixel belongs to the object of interest. These two tasks are interrelated and can assist the backbone to learn robust intermediate features for each other. Further, the segmentation result is used to guide the density regression. Specifically, to prevent background features from misleading the regressor, the weights of these features are reduced before being fed into the regressor. To achieve this, we generate a mask $M_{d}$ based on the predicted segmentation map:

(2) $$M_{d}=1\left(S_{\text{pred}}\geq 0.5\right)+\alpha 1\left(S_{\text{pred}}<0.5\right),$$

where α is the dampening factor and 1 is the indicator function. We set α as 0.1 and the generated mask $M_{d}$ is then applied on the intermediate features by point‐wise multiplication.

We down‐sample the $D_{\mathrm{gt}}$ by aggregating the density values to match the output size. The resulted learning target $D_{\text{target}}$ is further used in the generation of the ground‐truth segmentation map ($S_{\mathrm{gt}}$):

(3) $$S_{\mathrm{gt}}=1\left(D_{\text{target}}>\epsilon \right),$$

where *ϵ* is a density threshold and is set as 1 × 10^−3^ here.

##### Density branch & segmentation branch

The two branches in the model share a similar structure. They both consist of three convolutional layers: The first two have a kernel size of 3, while the last one has a kernel size of 1. These layers gradually reduce the number of channels of the extracted features from 512 to 1. The rectified linear unit (ReLU; Zeiler et al., 2013) is used as the activation function for the first two layers, with the activation function for the last layer of the two branches being different. The density branch is activated with the ReLU function to make sure every point on the output is non‐negative, whereas for the segmentation branch, the sigmoid (Han & Moraga, 1995) function is used to limit the range between 0 and 1.

#### 2.2.2 Loss function

Our overall loss function consists of two parts. First, we adopt the structural loss (*SL*) proposed by Rong and Li (2021) to supervise the density branch, defined as:

(4) $$\mathrm{SL}=\frac{1}{N}\sum_{i=1}^{N}\left(1-\text{SSIM}\left(\mathrm{Poo}l_{i}\left(D_{\text{pred}}\right),\mathrm{Poo}l_{i}\left(D_{\text{target}}\right)\right)\right),$$

where $D_{\text{pred}}$ represents the predicted density map, and *Pool* stands for average pooling which downsamples the map by a factor of $\frac{1}{2^{i-1}}$. *SSIM* is short for the Structural Similarity Index Measures (Wang et al., 2004) that can describe the similarity of two images, expressed as:

(5) $$\text{SSIM}\left(X,Y\right)=1-\frac{\left(2\mu_{X}\mu_{Y}+C_{1}\right)\left(2\sigma_{\mathrm{XY}}+C_{2}\right)}{\left(\mu_{X}^{2}+\mu_{Y}^{2}+C_{1}\right)\left(\sigma_{X}^{2}+\sigma_{Y}^{2}+C_{2}\right)},\;$$

where *μ* and *σ* denote mean and variance while $\sigma_{\mathrm{XY}}$ represents the covariance of *X* and *Y*. *C*_*1*_ and *C*_*2*_ are constants, set to 0.01 and 0.03 by default. The higher the SSIM index, the more similar the two images are. *N* is set as 3 following Wang et al.'s work.

The *SL* function improves the structural similarity between the prediction and the target by SSIM of high‐resolution maps, and the count accuracy is ensured by SSIM of the pooled density maps. Further, we make a minor change on the original loss function to improve counting accuracy, expressed as:

(6) $${\mathrm{SL}}^{*}=\frac{1}{N}\sum_{i=1}^{N}\left(1-\text{SSIM}\left(\mathrm{Poo}l_{i}\left(D_{\text{pred}}⊙S_{\mathrm{gt}}\right),\mathrm{Poo}l_{i}\left(D_{\text{target}}⊙S_{\mathrm{gt}}\right)\right)\right),$$

where *⊙* denotes point‐wise multiplication. This change eliminates the contribution to the loss value from points which have negligible values on the density maps. The original *SL* function pushes the value of each pixel on the predicted density map as close to the corresponding value on the target map as possible. However, in aerial images, if points are classified into two categories based on whether they have nonzero density values, the two classes are imbalanced. Most of the points are small values, or even zero, and since they are common the regressor will favor their estimation, meanwhile underestimating points with large density values. Noting large density values contribute most to the count, the counting accuracy will be harmed in unduly accommodating the low‐density regions. By masking points with small values, the regressor focus is on large density values and reduces their influence. During the inference stage, when integrated with the segmentation, we can safely discard the regressor's predictions on these points with small values and set them to 0.

The segmentation branch is supervised by the cross‐entropy (*CE*) loss function. We adjust it to minimize the impact of the imbalance in the number of positive and negative samples in the dataset:

(7) $$\mathrm{CE}=\frac{1}{M}\sum_{m=1}^{M}-\left(y_{m}\log\left(p_{m}\right)+h*\left(1-y_{m}\right)\;\log\left(1-p_{m}\right)\right)$$

where $y_{m}$ and $p_{m}$ is the corresponding value of location *m* in the image on the ground‐truth segmentation map and the predicted probability map. h is a constant, used for balancing the contribution of positive and negative samples to the loss value and is set as 0.5 in our experiments.

The final loss function is a weighted sum of the above two loss functions:

(8) $$\text{Loss}=SL^{*}+\mathrm{\lambda CE}$$

with *λ* set to 0.1 since the density estimation is the main task of the model.

#### 2.2.3 Model inference

Our model adopts a fully convolutional design, which means it has no strict size constraints on the input image. However, there are four max‐pooling layers with kernel size of 2 in the backbone structure, which may result in pixel dropout. To prevent this, the input image has to be enlarged to the smallest size divisible by 16. The output density map $D_{\text{out}}$ integrates the predictions from both branches and can be obtained by:

(9) $$D_{\text{out}}=D_{\text{pred}}⊙1\left(S_{\text{pred}}\geq 0.5\right)$$

#### 2.2.4 Experiments

We randomly split our dataset into three parts in a ratio of 3:1:1. The largest part serves as the training set, and the remaining parts are used for the purpose of validation and test, respectively. The detailed statistics of these three datasets are shown in Table 1. Notably, these datasets show drastic change in density distribution and all contain a few samples that are only backgrounds.

**TABLE 1:** Statistics of the training, validation, and test set.

Dataset	Number of images	L0	L1	L2	L3	L4	Total	Max	Average
Training set	446	118	140	137	34	17	87,654	2682	196
Validation set	146	35	61	34	14	2	23,918	1361	164
Test set	146	39	59	36	6	6	23,707	1580	162

*Note*: L0, L1, L2, L3, and L4 represent the number of images containing 0, 1–100, 101–500, 501–1000, and 1000+ penguins. Total gives the total number of penguins in the dataset, while Max and Average show the maximum and average number of penguins in one image in the dataset, respectively.

In our experiments, we adopt random cropping (256 × 256) and random horizontal flipping as data augmentation strategies for training the model. The parameters of the backbone are initialized with the VGG‐19 pretrained on ImageNet (Deng et al., 2009) and others are randomly initialized from a Gaussian distribution with a standard deviation of 0.01. We train the network for 600 epochs with a batch size of 16 using the Adam optimizer (Kingma & Ba, 2015). We fix the learning rate as 1e−5 and the weight decay as 1e−4, with validation starting after the 100th epoch. The model with the best performance on the validation set is used to report the final result on the test set.

For comparison, we also implement a Faster‐RCNN model, the detailed training process is provided in the Appendix A.

All experiments were conducted on a single 16 GB Tesla P100 GPU, with methods implemented with Pytorch. The whole training process takes approximately 3 h.

## 3 RESULTS

To evaluate our method, we use the mean absolute error (MAE) and root mean squared error (RMSE) metrics, defined as:

(10) $$\mathrm{MAE}=\frac{1}{N}\sum_{i=1}^{N}|C_{i}^{\text{pred}}-C_{i}^{\mathrm{gt}}|\;$$

(11) $$\text{RMSE}=\sqrt{\frac{1}{N}\sum_{i=1}^{N}{\left(C_{i}^{\text{pred}}-C_{i}^{\mathrm{gt}}\right)}^{2}}$$

where *N* is the total number of the images, $C_{i}^{\text{pred}}$ and $C_{i}^{\mathrm{gt}}$ is the predicted count and the ground‐truth count of *i*‐th image, respectively.

Mean absolute error gives the average error between predicted and target values, which can provide direct evidence of the accuracy of a model. However, it is not sufficiently sensitive to undesirable large errors. Therefore, RMSE is used as another evaluation metric since RMSE gives a relatively high weight to large errors. MAE and RMSE can be used jointly to diagnose the performance of a model. The larger the difference between them means the greater the variance in the individual errors in a dataset. It is unclear which one of these metrics is more important; hence, we simply define the model which has the lowest sum of MAE and RMSE on the validation data as the best model. This model's performance on the test set is shown in Table 2. To better illustrate our model's performance, we provide the results from a Faster‐RCNN model for comparison. In addition, separate average performance on images with different count levels, L0 (0), L1 (1–100), L2 (101–500), L3 (501–1000), and L4 (1000+), are also calculated. Overall, our model has an outstanding performance on this task and outperforms the Faster‐RCNN model in all aspects. It is also worth mentioning that the count error at the dataset level for our model is +186.6 (+0.8%) while for Faster‐RCNN is 4741 (+20.0%).

**TABLE 2:** Evaluation result of our model and the Faster‐RCNN on the test set.

Models	Overall		MAE					RMSE				
	MAE	RMSE	L0	L1	L2	L3	L4	L0	L1	L2	L3	L4
Our model	19.9	39.4	7.2	10.8	31.1	65.6	78.8	32.4	16.2	43.0	70.4	111.3
Faster‐RCNN	54.8	78.9	20.0	51.7	74.4	89.7	158.2	44.0	64.4	95.2	110.6	177.9

**FIGURE 4:**
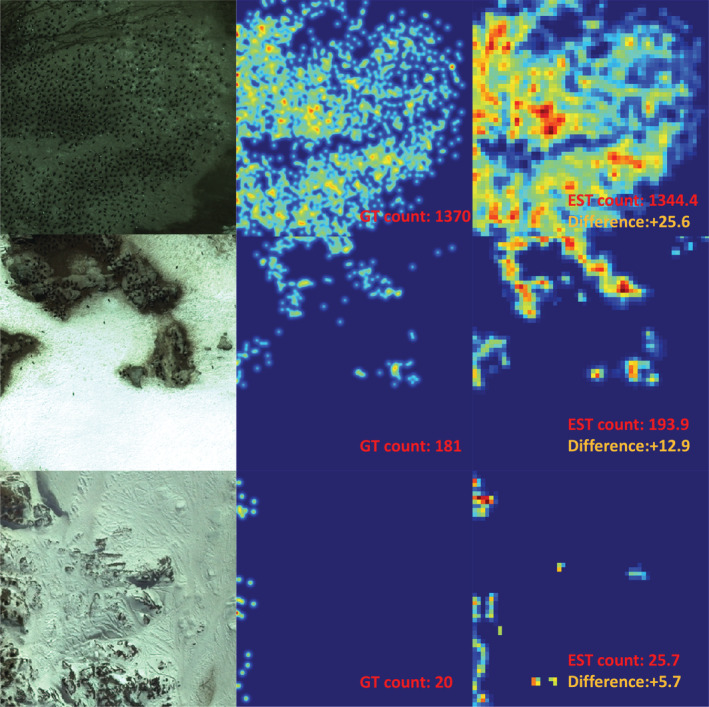
Some visualization results of the estimated density maps. The three images in each row, from left to right is the input, the Gaussian‐smoothed ground‐truth density map and the prediction. The corresponding count is given in the lower right corner of the density map. The difference between the ground‐truth and the estimated counts is highlighted.

Some of the estimated density maps are presented in Figure 4. Although the prediction's resolution is only one‐eighth the resolution of the generated ground‐true density map, it exhibits similar characteristics at the image level.

## 4 DISCUSSION

The algorithmic counting of objects in aerial images in ecological studies is currently dominated by detection algorithms. However, we have shown here that our model has various advantages over these methods.

Overall, our model has four main advantages over detection methods beyond markedly better performance on our data. First, our method is able to count extremely small objects. In the case of aerial images, the object of interest in an image is likely to be very small, especially for ecological surveys—in our case, only about 5 × 5 pixels. Our experiments show even the two‐stage detection algorithm Faster‐RCNN fails to detect most of the penguins. The reason is as follows: No matter what detection methods, a backbone structure is essential for extracting features. However, the current mainstream deep network structure, often used as the backbone, will downsample the image to a certain extent, for example, the downsampling ratio of VGG series is 16, while 32 for ResNet series (He et al., 2016). With a high downsampling ratio, the representation of a small object on the final feature maps may not be abundant enough for subsequent neural networks to predict the location and classification simultaneously. In contrast, our density estimation model only focuses on the counting of locations on the feature map instead of individuals, which provides better count accuracy.

Second, our model only requires point annotation, which means annotators need only to mark the same part of each object with a dot, quite similar to the way human counts. In contrast, detection algorithms require bounding box annotations and the quality of these will have a large impact on their performance (Russakovsky et al., 2015). For each object, a high‐quality bounding box is characterized as one with the smallest area but covers all the visible parts of that object. To create such annotation is laborious compared with point annotation, which only requires a point be drawn on the object and need not be especially accurate. This simplicity is exemplified in the generation of the ground‐truth density maps—the size of each penguin is not required, we need only apply the same normalized Gaussian kernel on every penguin.

Third, the density map estimation method can better handle objects located at the edge of the image. It is often the case that the images are of large size, with considering GPU memory constraints, researchers therefore have to crop them into digestible pieces for the deep learning networks. It is inevitable that some objects are also split into pieces, scattering them over several image patches. Such a situation results in a complex detection result where objects are undetected due to incomplete feature representations, or are repeatedly detected across multiple image patches. However, this will not pose a problem to the density estimation model, where the count of an object is not necessarily integer, thanks to the Gaussian smoothing. Hence, there will not produce redundant counts when summing up two nonoverlapping neighboring image patches.

Lastly, our model can utilize negative samples (images with zero counts) during training phase, which makes it more robust than the detection model when dealing with backgrounds. For some survey footage, there will be many images that are completely background, that is, no objects. However, detection algorithms cannot use them since they require every training sample to contain at least one object of interest. This is a fundamental short‐coming of the detection algorithms. Meanwhile, our model can fully use these images to improve its ability to differentiate the foreground and background. This also explains the large difference in performance of these two models on images of count level, L0.

In this work, we propose a CNN‐based density map estimation model to count extremely small penguins in aerial images, especially those acquired by manned aircraft surveys. Compared with the traditional two‐stage detection method, Faster‐RCNN (Ren et al., 2016), our model shows a significant improvement in counting accuracy when faced with small objects—specifically, marked improvements in MAE and RMSE of 63.7% and 50.1%, respectively, and at the dataset level where the count error is reduced by 19.2% to be almost zero. Furthermore, our model outperforms the Faster‐RCNN model over all levels of object density. Although the precise location of each object is not obtained with our model, it still indicates areas where objects may exist. In the event, object counting needs to be very precise—necessitating a human counter—our model aids by excluding regions that do not need detailed consideration. Overall, we expect our research to help researchers who are handling small objects with low resolution in aerial ecological surveys.

## AUTHOR CONTRIBUTIONS

**Yifei Qian:** Methodology (lead); writing – original draft (lead). **Grant R. W. Humphries:** Data curation (equal); writing – review and editing (equal). **Philip N. Trathan:** Data curation (equal); writing – review and editing (equal). **Andrew Lowther:** Writing – review and editing (equal). **Carl R. Donovan:** Methodology (supporting); supervision (lead); writing – review and editing (equal).

## CONFLICT OF INTEREST STATEMENT

The authors declare no competing interests.

## Figures and Tables

**FIGURE A1:**
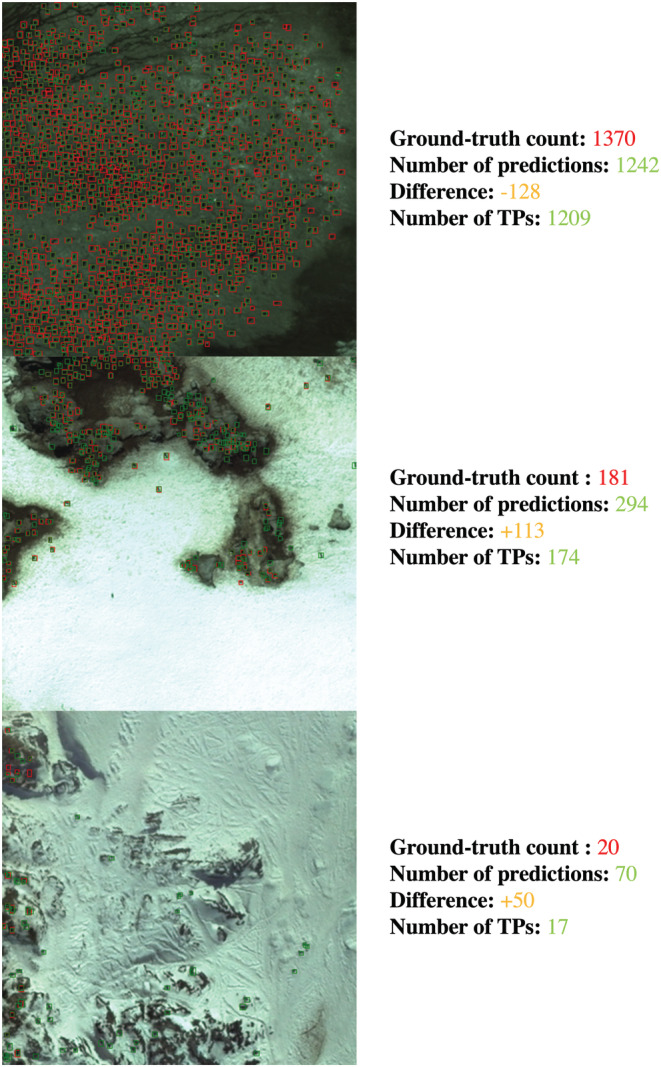
Visual examples of the Faster‐RCNN model when setting the IoU threshold to 0.3 and the confidence threshold to 0.2. In the left image, the ground‐truth bounding boxes are red, and the prediction boxes are green. The corresponding detailed statistic is given on the right.

## Data Availability

The data and the code used in this work are available at the Dryad Data and can be accessed via https://datadryad.org/stash/dataset/doi:10.5061/dryad.8931zcrv8. (Qian et al., 2023; Qian et al., 2022).

## APPENDIX A

### A.1 Specification of the Faster‐RCNN model

In this section, we provide the detailed training process of the Faster‐RCNN model and display full evaluation results.

### A.2 Experiments

Faster‐RCNN has many hyper‐parameters, in our experiments, most of them are kept in consistent with the original work (Ren et al., 2016)—we only highlight the differences here. The input images are enlarged by four times to ensure every object is larger than 16 × 16 pixels and detectable. The number of anchor boxes is reduced from 9 to 6 since the small variation in the size of objects. The size of these boxes are set as much as possible to match the size of the objects in the dataset—specifically the area of the anchor boxes are 16 × 16 and 24 × 24 with aspect ratios of 0.9, 1, 1.1.

For fair comparison, we use the pretrained VGG‐19 as backbone in the Faster‐RCNN model. The data augmentation technique used here is only random horizontal flipping. The batch size is set as 1. A total of 25 epoch are trained with the stochastic gradient descent (SGD) optimizer (Ruder, 2017). The initial learning rate is set as 1e−3 and decays to 1e−4 at the twelfth epoch. Only images without negative samples are used for training, and the whole training process takes around 5 h to complete.

### A.3 Results

Different to the density map estimation method, we define the model with the highest 11‐points interpolated average precision (*AP*) score on the validation set as the best detection model. The *AP* is computed from 11 recall levels and can be expressed as:

(12)
$$\mathrm{AP}=\frac{1}{11}\sum_{r\in \left\{0.0,0.1,\ldots 1.0\right\}}p_{\text{interp}}\left(r\right),$$

(13)
$$p_{\text{interp}}\left(r\right)=\underset{r^{\prime }\geq r}{\max\;}p\left(r^{\prime }\right),\;$$

where p is the precision and r is the recall. $p_{\text{interp}}\left(r\right)$ represents the maximum precision for any recall $r^{\prime }\geq r$. The definition of precision and recall is given below:

(14)
$$\text{Precision}=\frac{\mathrm{TP}}{\mathrm{TP}+\mathrm{FP}},\;$$

(15)
$$\text{Recall}=\frac{\mathrm{TP}}{\mathrm{TP}+\mathrm{FN}},\;$$

where TP is the true positive, FP is the false positive, and FN is the false negative.

Here, we report the results of the best detection model on the test set. The detection results are varied with the intersection over union (IoU) threshold and the confidence threshold. The IoU threshold is fixed as 0.3 in our experiments, and we only adjust the confidence threshold. For better understanding, we still present the performance of the detection model with MAE and RMSE metrics and the full results are displayed in Table A1.

**TABLE A1:** Results of the Faster‐RCNN model on the entire test set. The IoU threshold is fixed as 0.3.

Confidence level	MAE	RMSE	Count error	Precision
0.1	97.97	123.55	13,387 [56.47%]	0.56
0.2	54.79	78.93	4741 [20.00%]	0.68
0.3	53.02	103.24	−1107 [−4.67%]	0.75
0.4	64.21	151.27	−6405 [−27.02%]	0.80
0.5	92.63	226.50	−12,525 [−52.83%]	0.83
0.6	131.42	301.72	−18,907 [−79.75%]	0.84

We visualize some detection results in Figure A1 under the confidence level of 0.2. As shown in the graph, the detection model gives precise location of each prediction; however, it is very vulnerable to the complex scenes and varied count levels.
